# Supplementary material for: Sea temperature and pollution are associated with infectious disease mortality in short-beaked common dolphins
Source: Commun Biol. 2025 Apr 11;8:557. doi: 10.1038/s42003-025-07858-7 (PMC11992094; doi:10.1038/s42003-025-07858-7)
Supplement: Supplementary file 1 — Supplementary Information [file 42003_2025_7858_MOESM1_ESM.pdf]

Supplementary Information

Figures

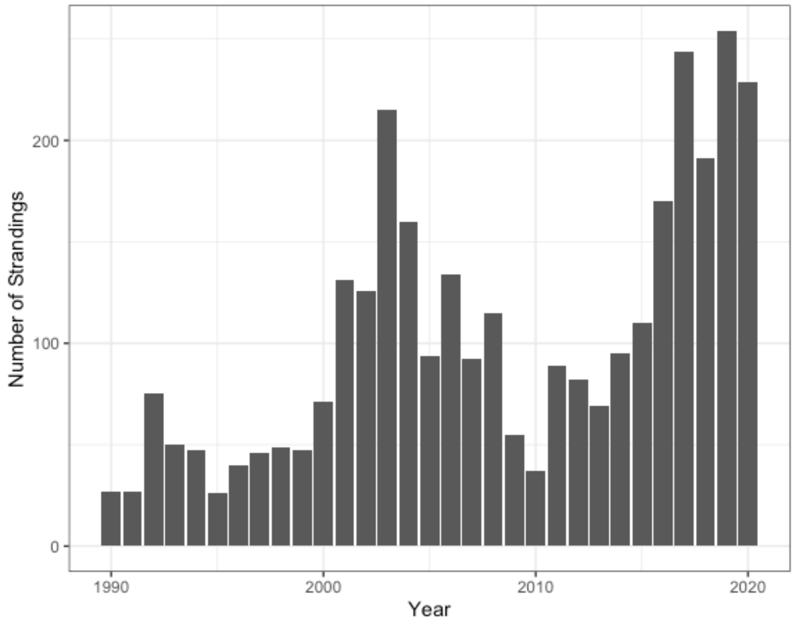

Supplementary Figure 1: Yearly totals of the overall number of reported strandings for short-beaked common dolphins

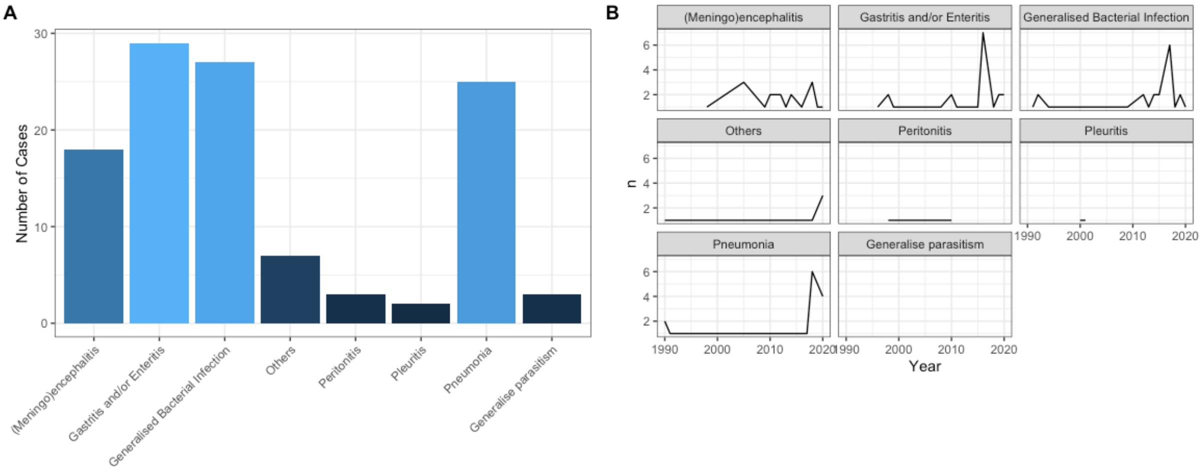

Supplementary Figure 2: (A) Number of cases in each infectious disease category (B) Yearly frequencies for each infectious disease category for short-beaked common dolphin that underwent necropsy.

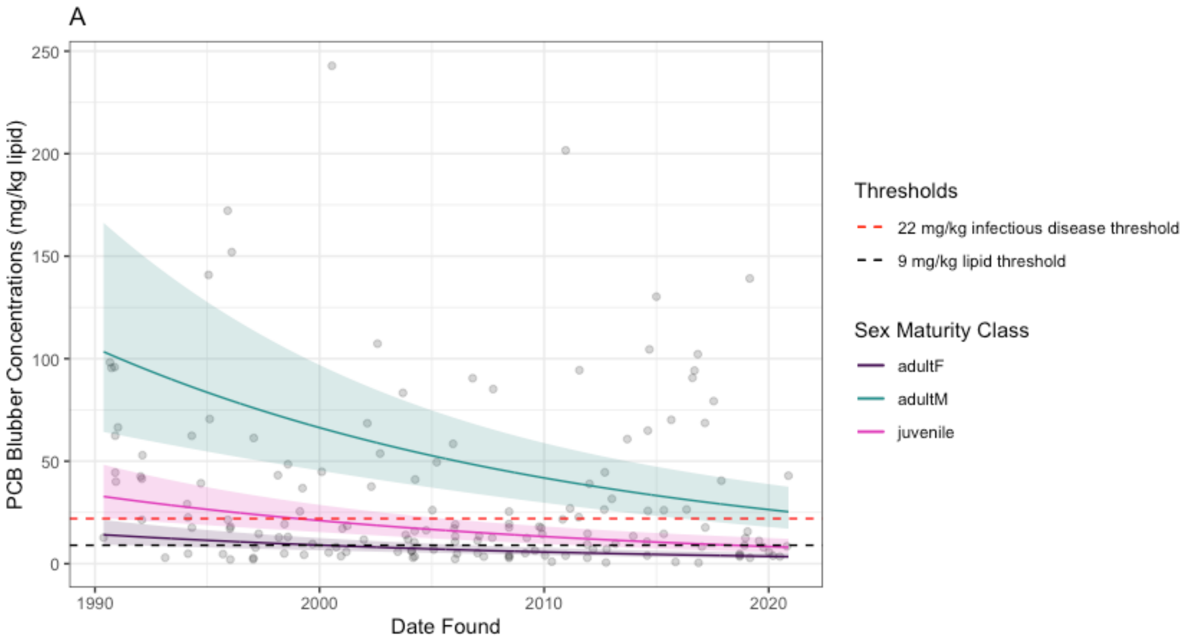

Supplementary Figure 3: Modelled PCB blubber concentrations (mg/kg lipid) for each of the age and sex classes. The shaded ribbons represent the 95% confidence intervals. This is an adjusted version of Figure 4 from the main text (without the limitation on the y-axis).

Tables

Supplementary Table 1: Results of the Mann-Kendell trend tests to assess temporal trends in causes of mortality

Cause of Death	Tau (τ)	2-sided p-value
Trauma	0.0288	0.837
Others	0.165	0.207
Live Stranding	0.22	0.115

Supplementary Table 2: Variable combinations, Δ AIC values and model weights for the models that were averaged to obtain the final generalised linear model (GLM) used to investigate relationships between SSTs (sea surface temperature), PCB (polychlorinated biphenyl) blubber concentrations and infectious disease mortality.

Age Sex Class	Latitude	Longitude	Body condition	Mean SST	PCBs	Mean SST * PCBs	Δ AIC	Weight
-	+	-	-	+	+	-	0	0.11
-	-	-	-	+	+	-	0.37	0.092
-	+	-	+	+	+	-	1.42	0.054
-	+	+	-	+	+	-	1.42	0.054
-	-	+	-	+	+	-	1.48	0.052
-	-	-	+	+	+	-	1.58	0.05
-	+	-	-	+	+	+	1.96	0.041

Supplementary Table 3: Model averaged coefficients for SST derived for two OSPAR areas, variables were centered and scaled.

	Estimate	Std. Error	Adjusted SE	z value	Pr(>|z|)
(Intercept)	-6.28	4.71	4.73	1.33	0.18
Latitude	3.42	4.33	4.35	0.79	0.43
Monthly mean SST	1.06	0.95	0.96	1.10	0.27
PCB concentration	0.02	0.01	0.01	2.36	<0.05*
Body condition	-0.06	0.15	0.15	0.42	0.68
Longitude	0.00	0.05	0.05	0.10	0.92
Monthly mean SST :PCB concentration	0.00	0.01	0.01	0.05	0.96

Supplementary Table 4: Results of model with larger dataset that did not include PCB concentration with SSTs calculated using two OSPAR areas. Variables were centered and scaled.

	Estimate	Std. Error	Adjusted SE	z value	Pr(>|z|)
(Intercept)	-0.61	0.50	0.50	1.23	0.22
Adult Males	-0.26	0.37	0.37	0.71	0.48
Juvenile	-0.50	0.49	0.49	1.02	0.31
Latitude	1.04	0.17	0.17	6.01	<0.05*
Body condition	-0.12	0.18	0.18	0.65	0.51
Monthly mean SST	0.84	0.14	0.14	6.02	<0.05*
Longitude	0.03	0.08	0.08	0.40	0.69

Supplementary Table 5: Results of model with larger dataset that did not include PCB concentration with SSTs calculated using four OSPAR areas. Variables were centered and scaled.

	Estimate	Std. Error	Adjusted SE	z value	Pr(>|z|)
(Intercept)	-24.129	10.779	10.808	2.233	0.026
Adult Males	-0.508	0.399	0.400	1.271	0.204
Juveniles	-0.966	0.391	0.392	2.462	0.014
Latitude	0.427	0.210	0.211	2.024	0.043
Body condition	1.172	1.994	1.999	0.587	0.557
Monthly mean SST	-0.850	0.955	0.957	0.889	0.374
Juveniles	0.304	0.047	0.047	6.452	<0.05*
Latitude:Longitude	-0.017	0.039	0.039	0.439	0.661

Supplementary Table 6: Model averaged coefficients for SST derived for two OSPAR areas, to derive PCB threshold for significance.

	Estimate	Std. Error	Adjusted SE	z value	Pr(>|z|)
(Intercept)	-6.02	4.33	4.35	1.39	0.17
Latitude	0.05	0.08	0.08	0.69	0.49
Monthly mean SST	0.15	0.06	0.06	2.46	<0.05*
PCB threshold	0.92	0.42	0.42	2.17	<0.05*
Body condition	-0.31	0.86	0.87	0.36	0.72
Longitude	0.03	0.10	0.10	0.33	0.74

Supplementary Table 7: Model averaged coefficients for the averaged logistic regression model used to investigate relationships between SSTs (sea surface temperature), PCB (polychlorinated biphenyl) blubber concentrations and live strandings.SST was derived for four OSPAR areas.

	Estimate	Std. Error	Adjusted SE	z value	Pr(>|z|)
(Intercept)	-30.59	7.05	7.12	4.30	0.00
Latitude	0.55	0.12	0.13	4.41	<0.05*
Longitude	0.11	0.20	0.20	0.54	0.59
Monthly mean SST	0.09	0.13	0.14	0.64	0.52
PCB Concentration	0.02	0.04	0.04	0.51	0.61
Monthly mean SST :PCB concentration	0.00	0.00	0.00	0.47	0.64
Body condition	-0.02	0.52	0.53	0.04	0.97

Supplementary Table 8: Model averaged coefficients for temporal trend of PCBs.

	Estimate	Std. Error	Adjusted SE	z value	Pr(>|z|)
(Intercept)	-47.02	11.24	11.40	4.13	<0.05*
Adult Males	-2.05	1.15	1.17	1.76	0.08
Juvenile	-2.65	0.95	0.96	2.76	<0.05*
Latitude	43.91	11.01	11.17	3.93	<0.05*
Body condition	-1.06	0.36	0.37	2.89	<0.05*
Monthly mean SST	3.86	1.22	1.24	3.11	<0.05*
PCB Concentration	1.70	0.71	0.72	2.36	<0.05*
Longitude	0.06	0.16	0.16	0.38	0.71

Supplementary Table 9: Model averaged coefficients for temporal trend of PCBs in adult males

	Estimate	Std. Error	Adjusted SE	z value	Pr(>|z|)
(Intercept)	5.22	2.38	2.44	2.14	<0.05*
Date.Found	0.00	0.00	0.00	1.50	0.13
Latitude	-0.04	0.07	0.07	0.57	0.57
Rel.body.wt	-0.53	1.11	1.15	0.46	0.64
Longitude	0.04	0.16	0.17	0.23	0.82

Supplementary Table 10: Model averaged coefficients for temporal trend of PCBs in adult females

	Estimate	Std. Error	Adjusted SE	z value	Pr(>|z|)
(Intercept)	11.57	1.68	1.72	6.74	<0.05*
Date.Found	0.00	0.00	0.00	2.95	<0.05*
Latitude	-0.15	0.03	0.03	4.52	<0.05*
Rel.body.wt	-0.45	0.55	0.56	0.81	0.42
Longitude	0.03	0.36	0.37	0.09	0.93
Latitude:Longitude	0.00	0.02	0.02	0.01	1.00

Supplementary Table 11: Model averaged coefficients for temporal trend of PCBs in juveniles

	Estimate	Std. Error	Adjusted SE	z value	Pr(>|z|)
(Intercept)	17.64	13.10	13.46	1.31	0.19
Date.Found	0.00	0.00	0.00	2.01	<0.05*
Latitude	-0.27	0.26	0.27	1.01	0.31
Rel.body.wt	-2.63	1.19	1.22	2.15	<0.05*
Longitude	-0.39	3.85	3.95	0.10	0.92
Latitude:Longitude	0.05	0.15	0.16	0.31	0.76

Supplementary Table 12: Percentage of non-detects for each congener (n=217)

Congener	Percentage of non-detects
CB.18	35.94
CB.28	35.02
CB.31	49.31
CB.44	8.76
CB.47	3.69
CB.49	11.98
CB.52	1.38
CB.66	1.84
CB.101	0.00

Congener	Percentage of non-detects
CB.105	1.84
CB.110	4.61
CB.118	0.00
CB.128	0.92
CB.138	0.00
CB.141	7.83
CB.149	0.00
CB.151	0.92
CB.153	0.00
CB.156	1.84
CB.158	3.69
CB.170	0.00
CB.180	0.00
CB.183	0.92
CB.187	0.00
CB.194	0.92
