# Supplementary material for: Sea temperature and pollution are associated with infectious disease mortality in short-beaked common dolphins
Source: Commun Biol. 2025 Apr 11;8:557. doi: 10.1038/s42003-025-07858-7 (PMC11992094; doi:10.1038/s42003-025-07858-7)
Supplement: Supplementary file 2 — Reporting Summary [file 42003_2025_7858_MOESM2_ESM.pdf]

nature research

Corresponding author(s): Rosie Williams

Last updated by author(s): 02/14/2025

Reporting Summary

Nature Research wishes to improve the reproducibility of the work that we publish. This form provides structure for consistency and transparency in reporting. For further information on Nature Research policies, see our Editorial Policies and the Editorial Policy Checklist.

Statistics

For all statistical analyses, confirm that the following items are present in the figure legend, table legend, main text, or Methods section.

n/a	Confirmed	
☐	☒	The exact sample size (n) for each experimental group/condition, given as a discrete number and unit of measurement
☐	☒	A statement on whether measurements were taken from distinct samples or whether the same sample was measured repeatedly
☐	☒	The statistical test(s) used AND whether they are one- or two-sided<br>*Only common tests should be described solely by name; describe more complex techniques in the Methods section.*
☐	☒	A description of all covariates tested
☐	☒	A description of any assumptions or corrections, such as tests of normality and adjustment for multiple comparisons
☐	☒	A full description of the statistical parameters including central tendency (e.g. means) or other basic estimates (e.g. regression coefficient) AND variation (e.g. standard deviation) or associated estimates of uncertainty (e.g. confidence intervals)
☐	☒	For null hypothesis testing, the test statistic (e.g. F, t, r) with confidence intervals, effect sizes, degrees of freedom and P value noted<br>*Give P values as exact values whenever suitable.*
☒	☐	For Bayesian analysis, information on the choice of priors and Markov chain Monte Carlo settings
☒	☐	For hierarchical and complex designs, identification of the appropriate level for tests and full reporting of outcomes
☒	☐	Estimates of effect sizes (e.g. Cohen's d, Pearson's r), indicating how they were calculated

Our web collection on statistics for biologists contains articles on many of the points above.

Software and code

Policy information about availability of computer code

Data collection	No software was used for data collection
Data analysis	Custom code was used within R version 4.0 to analyze that data in this study. The R code that supports the findings of this study will be made available via zenodo.

For manuscripts utilizing custom algorithms or software that are central to the research but not yet described in published literature, software must be made available to editors and reviewers. We strongly encourage code deposition in a community repository (e.g. GitHub). See the Nature Research guidelines for submitting code & software for further information.

Data

Policy information about availability of data

All manuscripts must include a data availability statement. This statement should provide the following information, where applicable:

- Accession codes, unique identifiers, or web links for publicly available datasets
- A list of figures that have associated raw data
- A description of any restrictions on data availability

Raw data supporting the results are available from the from the British Oceanographic Data Centre (BODC). *These details will be updated to provide exact details and DOIs ahead of publication.

Field-specific reporting

Please select the one below that is the best fit for your research. If you are not sure, read the appropriate sections before making your selection.

☐ Life sciences ☐ Behavioural & social sciences ☒ Ecological, evolutionary & environmental sciences

For a reference copy of the document with all sections, see nature.com/documents/nr-reporting-summary-flat.pdf

Ecological, evolutionary & environmental sciences study design

All studies must disclose on these points even when the disclosure is negative.

Study description	Blubber samples were opportunistically sampled from stranded, deceased marine mammals. Pollutant concentrations in blubber samples were analyzed. Samples were prioritized for analysis according to their level of decomposition. The samples that were analyzed were compared with the wider strandings dataset to confirm that they were a representative sample.
Research sample	This study used an existing dataset held by the Cetacean Strandings Investigation Programme, which documents incidences of marine mammal strandings in the United Kingdom. Information on each stranding, including, location, species, date, sex, length, weight and cause of death, was recorded.
Sampling strategy	Sampling was opportunistic. Therefore, sample sizes were determined by the dataset available. Data exploration and statistical analyses confirmed that the sample size was sufficient.
Data collection	The data were recorded by a number of pathologists and toxicologists over the 30 year study period.
Timing and spatial scale	The data have been continuously collected throughout the study period (1990-2018). The spatial scale covers the coastline of the United Kingdom.
Data exclusions	A small number of individuals were excluded from statistical modeling due to missing data (i.e. sex, age class, blubber thickness)
Reproducibility	No experiments were carried out but all code will be available on zenodo to re-run the analyses and data are available from British Oceanographic Data Centre.
Randomization	This is not relevant to our study as no experiments were carried out and sampling was opportunistic.
Blinding	Blinding was not relevant to the study as no experiments were carried out and data acquisition was opportunistic.

Did the study involve field work? ☐ Yes ☒ No

Reporting for specific materials, systems and methods

We require information from authors about some types of materials, experimental systems and methods used in many studies. Here, indicate whether each material, system or method listed is relevant to your study. If you are not sure if a list item applies to your research, read the appropriate section before selecting a response.

Materials & experimental systems

n/a	Involved in the study
☒	☐ Antibodies
☒	☐ Eukaryotic cell lines
☒	☐ Palaeontology and archaeology
☒	☐ Animals and other organisms
☒	☐ Human research participants
☒	☐ Clinical data
☒	☐ Dual use research of concern

Methods

n/a	Involved in the study
☒	☐ ChIP-seq
☒	☐ Flow cytometry
☒	☐ MRI-based neuroimaging
